# Association between dietary folate intake and bone mineral density in a diverse population: a cross-sectional study

**DOI:** 10.1186/s13018-023-04188-4

**Published:** 2023-09-14

**Authors:** Zitian Zheng, Huanhuan Luo, Wennan Xu, Qingyun Xue

**Affiliations:** 1 Department of Orthopedics, Beijing Hospital, National Center of Gerontology, Institute of Geriatric Medicine, Chinese Academy of Medical Sciences, NO.1 Da Hua Road, DongDan, Beijing, 100730 People’s Republic of China; 2 Peking University Fifth School of Clinical Medicine, Beijing, People’s Republic of China; 3 Department of Nursing, Beijing Hospital, National Center of Gerontology, Institute of Geriatric Medicine, Chinese Academy of Medical Science, Beijing, People’s Republic of China; 4 Graduate School, Peking Union Medical College, Beijing, People’s Republic of China; 5 Department of Orthopedics, Sun Yat-sen Memorial Hospital, Sun Yat-sen University, Guangzhou, People’s Republic of China

**Keywords:** Dietary folate intake, Bone mineral density, Nonlinear relationship, Cross-sectional

## Abstract

**Background:**

Osteoporosis is a major public health problem, yet the association between dietary folate intake and bone health has been rarely studied. This study aimed to investigate the relationship between dietary folate intake and bone mineral density (BMD) in the general population of the USA.

**Methods:**

Utilizing data from the National Health and Nutrition Examination Survey, dietary folate intake was gauged through 24-h dietary recall and BMD was determined via dual-energy X-ray absorptiometry. Multivariate linear regression models and generalized additive models were employed for correlation analysis.

**Results:**

The study incorporated 9839 participants (48.88% males, aged 20–85 years, mean age: 47.62 ± 16.22). The average dietary folate intake stood at 401.1 ± 207.9 μg/day. And the average total femur, femoral neck, trochanter, intertrochanter, and lumbar BMD were 0.98 ± 0.16 g/cm^2^, 0.84 ± 0.15 g/cm^2^, 0.73 ± 0.13 g/cm^2^, 1.16 ± 0.19 g/cm^2^, and 1.03 ± 0.15 g/cm^2^, respectively. The higher quartiles of dietary folate intake directly correlated with increased total femoral, femoral neck, intertrochanteric, and lumbar BMD (*P* for trend = 0.003, 0.016, < 0.001, and 0.033, respectively). A consistent positive association between folate intake and BMD across age groups was observed, with significant findings for individuals over 80 years and non-Hispanic whites. Physical activity level and serum 25-hydroxyvitamin D levels influenced the association, with an optimal daily folate intake of 528–569 µg recommended for postmenopausal women.

**Conclusion:**

In summary, our study reveals a significant positive association between dietary folate intake and BMD, across different age groups and particularly among individuals over 80 years old. Non-Hispanic whites benefit the most from increased folate intake. Physical activity level and serum 25-hydroxyvitamin D levels interact with this association. Screening and early intervention for osteoporosis may be essential for individuals with low dietary folate intake.

## Introduction

Osteoporosis, hallmarked by diminished BMD, escalates the risk of severe fractures within the hip, vertebrae, and pelvis [1, 2]. Such fractures constitute a primary cause of morbidity and mortality within the geriatric population and engender considerable medical and economic strain on families and the larger society [3, 4].

Folate, a water-soluble vitamin, has been recognized for its crucial role in lipid metabolism regulation and antioxidant activity [5]. The pathogenesis of osteoporosis is underpinned by oxidative stress [6, 7], where an escalation in bone resorption [8], a decline in osteoblast activity [9], and amplified osteoblast and osteocyte apoptosis [10], collectively contribute to decreased BMD. The potential of natural antioxidants, such as folate [11], and antioxidant supplements to augment BMD and mitigate fracture risk [12, 13] has spurred interest in understanding the effects of dietary antioxidants on BMD, a critical cornerstone of preventive strategies [13–15]; the existing body of knowledge bears conspicuous gaps.

The preponderance of current BMD literature is disproportionately skewed toward postmenopausal women or the elderly [16, 17]. However, the emerging issue of BMD deterioration in younger, middle-aged adults resulting from increasing unhealthy lifestyle trends and escalating severity of environmental pollution in the industrial era calls for urgent academic attention [18, 19]. Furthermore, current research typically concentrates on BMD exploration within either the lumbar spine [20] or femur alone [21]. Given the possibility of BMD inconsistency across these regions [22], reliance on single-region evaluation may yield incomplete findings. Additionally, synthesizing multiple studies to infer the effects of dietary folate on BMD in the spine and femur may introduce bias due to the population heterogeneity inherent in these studies. In view of the population's complex heterogeneity [17], there is a compelling need for stratified studies based on large samples.

This study seeks to fill these knowledge voids by investigating the influence of dietary folate on BMD across various body regions, including the total femur, femoral neck, trochanter, intertrochanter, and lumbar spine, within a broad cross section of the US population, extending beyond the elderly demographic. Employing a substantial sample size from the US population, this research also aims to evaluate BMD stratified by variables such as menopausal status, gender, 25(OH)D, physical activity, age, and race.

## Materials and methods

### Study population

The present investigation utilized data harvested from the National Health and Nutrition Examination Survey (NHANES), an extensive research endeavor managed by the National Center for Health Statistics (NCHS). The NHANES amalgamates interviews, physical examinations, and laboratory assessments to yield vital health statistics for the population of the USA [23, 24]. Following the exclusion of participants who had incomplete data regarding dietary folate intake and BMD measurements, the study incorporated a cohort of 9839 individuals. These participants were selected from four NHANES cycles [2005–2010, 2013–2014] for the multi-site BMD analysis, encompassing the total femur, femoral neck, trochanter, intertrochanter, and the lumbar spine.

**Fig. 1:**
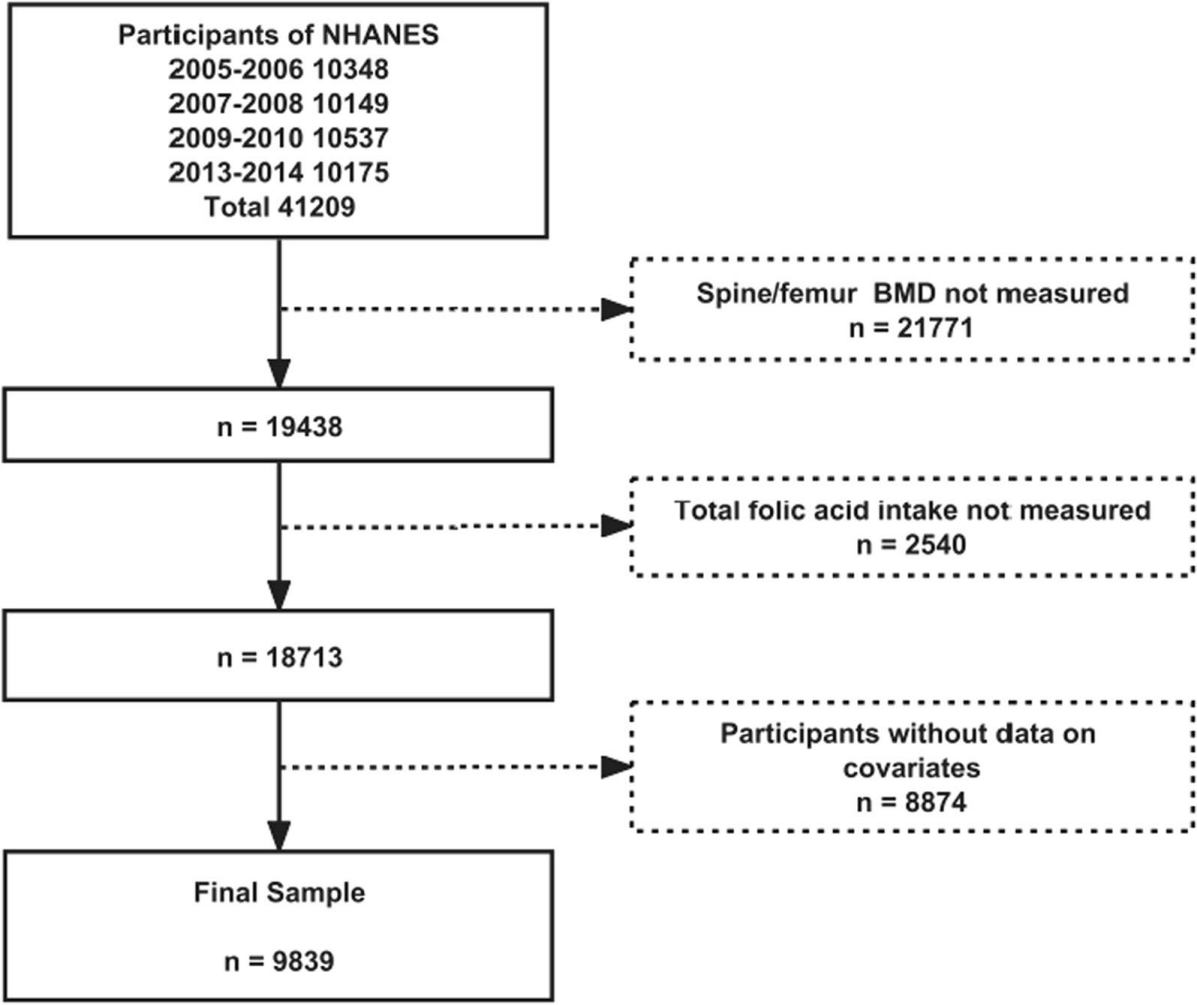
Flowchart of participant selection

The comprehensive selection process for our study participants is delineated in Fig. 1. From the 2005–2006, 2007–2008, 2009–2010, and 2013–2014 cycles of NHANES, we initially identified 41,209 participants. Thereafter, subjects with incomplete data regarding femoral and lumbar spine bone mineral density (BMD) (*n *= 21,771) or dietary folate intake (*n *= 2540) were excluded. Furthermore, we removed participants with missing covariate data such as age and gender (*n *= 8874). Consequently, a total of 9839 individuals were incorporated into the final analysis.

### Assessment of dietary folate intake

Data regarding the intake of folate and other nutrients were compiled through 24-h dietary recall interviews, conducted by trained nutrition professionals. Participants were requested to reminiscence their food and beverage consumption over the prior 24-h period, detailing the timing, quantity, and nature of each item ingested, in addition to the brand name of any processed foods. The nutrient composition of recalled food items was determined employing the Argenfoods nutrient composition database and the United States Department of Agriculture (USDA) database. Nutrient intake data were computed utilizing the Automated Multiple-Pass Method (AMPM) (http://www.ars.usda.gov/ba/bhnrc/fsrg). Numerous studies [25–27] have confirmed the effectiveness of the AMPM in accurately estimating nutrient intake in adults. Folate intake was assessed in micrograms per day (μg/day).

### BMD measurement

BMD indices were ascertained at various sites (lumbar spine and total femur, femoral neck, trochanter, and intertrochanter) deploying the Hologic QDR 4500A fan-beam densitometer (Hologic Inc., Bedford, MA, USA) and the dual-energy X-ray absorptiometry (DXA) technique. The average BMD of the first through fourth lumbar vertebrae was employed to determine lumbar spine BMD. All DXA scan data were analyzed using Hologic APEX v3.0 software. In this study, the BMD indices at the total femur, femoral neck, trochanter, intertrochanter, and lumbar spine were evaluated.

### Other covariates

Considering our study's significant sample size, a meticulous statistical analysis was required, necessitating a comprehensive adjustment for appropriate covariates. Various clinical data, identified as covariates, were incorporated due to their potential impact on the correlation between dietary folate intake and bone mineral density (BMD). These covariates were partitioned into four distinct categories. The first category, demographic data, included variables such as age, gender, ethnicity (Mexican American, other Hispanic, non-Hispanic white, non-Hispanic black, and other races), the ratio of family income to poverty (Poverty Income Ratio, PIR), and marital status (Married/Cohabiting, Widowed/Divorced/Separated, or Never Married). The second category, laboratory data, composed of serum 25-hydroxyvitamin D [25(OH)D] and serum cotinine concentrations. The third category, examination data, included the body mass index (BMI). Lastly, the questionnaire data encapsulated physical activity, alcohol consumption, and hypertension status.

We further categorized the ratio of family income to poverty into three levels: low income (≤ 1.30), moderate income (1.31–2.40), and high income (> 2.4) [28]. Physical activity (PA) was quantified using the metabolic equivalent (MET) score as outlined on the official NHANES website. Subsequently, we defined physical activity as the total MET minutes per week, aggregated across all activity-related queries, and divided into very low PA (VLPA) (< 150 MET-min/week), low PA (LPA) (150–960 MET-min/week), medium PA (MPA) (961–1800 MET-min/week), and high PA (HPA) (> 1800 MET-min/week) [28]. Hypertension and alcohol consumption status were ascertained based on self-reported participant information. Through these comprehensive adjustments, our study sought to mitigate possible bias and enhance the precision of the resulting data.

### Statistical analysis

The baseline characteristics of study participants were presented in terms of mean ± standard deviation (SD) for continuous variables and percentages for categorical variables. The study divided dietary folate intake into quartiles, and the consequent variances among these quartiles were evaluated using analysis of variance (ANOVA) for continuous variables and Chi-square analysis for categorical variables.

The relationship between dietary folate intake and bone mineral density (BMD) was assessed through multivariable linear regression models. In adherence to the Strengthening the Reporting of Observational Studies in Epidemiology guidelines [29], four distinct models were constructed. Model 1 incorporated no adjustment for covariates, Model 2 adjusted for fundamental demographic variables, Model 3 extended the adjustment to include variables that have previously demonstrated a strong correlation with BMD, and Model 4 made an additional adjustment for the biological indicator serum 25-hydroxyvitamin D to control for potential nutritional confounders impacting BMD.

The dose–response relationship between dietary folate intake and BMD was further dissected through generalized additive model (GAM) smoothing curve fitting. This method facilitates the examination of nonlinear relationships between outcome variables and risk factors, thereby assisting in the detection of threshold effects [30]. If a nonlinear correlation was discernible, a two-piecewise linear regression model (or a segmented regression model) was utilized to determine the threshold effect. A two-step recursive methodology was then employed to identify the breakpoint (K) that links the segments, relying on a maximum likelihood model [31, 32].

Acknowledging the heightened vulnerability of women, especially postmenopausal women, to osteoporosis and diminished BMD [33–35], we conducted subgroup analysis stratified by premenopausal women, postmenopausal women, and men. Owing to the large and diverse sample size of our study, we further stratified the analyses based on race, physical activity, and serum 25(OH)D levels. We then constructed smoothed curve-fitting models in each population to explore the consistency of associations between dietary folate intake and BMD across populations. In the final stratified smoothed curve-fitting plots, due to the considerable quantity of strata, only estimated values were displayed, with confidence intervals omitted [36].

All statistical analyses were performed using the EmpowerStats statistical software (www.empowerstats.com) and R software. The threshold for statistical significance was set at a two-sided *P* value of < 0.05.

## Results

### Participant characteristics

Table 1 presents the demographic attributes of the study's participants. The study incorporated 9839 individuals, with a mean age of 47.62 ± 16.22 years. The male participants represented approximately 48.88% of the total sample. Non-Hispanic whites comprised 47.08% of the studied population, and around 63.95% of the subjects reported being married.

**Table 1:** Characteristics of the participant

Characteristics	Total population (*n *= 9839)	Dietary folate intake (quartile)				*P* value
		Q1 (*n *= 2455)	Q2 (*n *= 2463)	Q3 (*n *= 2455)	Q4 (*n *= 2466)	
Age	47.62 ± 16.22	48.95 ± 16.49	49.03 ± 16.47	46.81 ± 15.83	45.69 ± 15.84	< 0.001
Gender						< 0.001
Male	4809 (48.88%)	1565 (63.75%)	1348 (54.73%)	1132 (46.11%)	764 (30.98%)	
Female	5030 (51.12%)	890 (36.25%)	1115 (45.27%)	1323 (53.89%)	1702 (69.02%)	
Race						< 0.001
Mexican American	1845 (18.75%)	455 (18.53%)	486 (19.73%)	467 (19.02%)	437 (17.72%)	
Other Hispanic	902 (9.17%)	216 (8.80%)	236 (9.58%)	236 (9.61%)	214 (8.68%)	
Non-Hispanic white	4632 (47.08%)	1022 (41.63%)	1143 (46.41%)	1166 (47.49%)	1301 (52.76%)	
Non-Hispanic black	1853 (18.83%)	629 (25.62%)	467 (18.96%)	431 (17.56%)	326 (13.22%)	
Other race	607 (6.17%)	133 (5.42%)	131 (5.32%)	155 (6.31%)	188 (7.62%)	
Family income-poverty ratio						< 0.001
< 1.3	2518 (27.52%)	767 (34.01%)	599 (26.19%)	576 (25.12%)	576 (24.87%)	
1.3–2.4	3426 (37.44%)	890 (39.47%)	889 (38.87%)	875 (38.16%)	772 (33.33%)	
≥ 2.4	3207 (35.05%)	598 (26.52%)	799 (34.94%)	842 (36.72%)	968 (41.80%)	
Marital status						< 0.001
Married/cohabiting	6288 (63.95%)	1429 (58.23%)	1546 (62.82%)	1641 (66.87%)	1672 (67.86%)	
Widowed/divorced/separated	1908 (19.40%)	591 (24.08%)	540 (21.94%)	412 (16.79%)	365 (14.81%)	
Never married	1637 (16.65%)	434 (17.69%)	375 (15.24%)	401 (16.34%)	427 (17.33%)	
Body mass index						< 0.001
< 18.5	3096 (31.56%)	727 (29.71%)	745 (30.35%)	758 (30.93%)	866 (35.26%)	
18.5–30	3656 (37.27%)	865 (35.35%)	931 (37.92%)	926 (37.78%)	934 (38.03%)	
> 30	3057 (31.17%)	855 (34.94%)	779 (31.73%)	767 (31.29%)	656 (26.71%)	
Hypertension						< 0.001
No	7482 (81.15%)	1789 (78.57%)	1830 (79.77%)	1921 (82.98%)	1942 (83.20%)	
Yes	1738 (18.85%)	488 (21.43%)	464 (20.23%)	394 (17.02%)	392 (16.80%)	
Physical activity						< 0.001
VLPA	2259 (23.42%)	716 (29.62%)	622 (25.71%)	508 (21.12%)	413 (17.17%)	
LPA	2165 (22.44%)	540 (22.34%)	579 (23.94%)	559 (23.24%)	487 (20.24%)	
MPA	1307 (13.55%)	321 (13.28%)	315 (13.02%)	326 (13.56%)	345 (14.34%)	
HPA	3916 (40.59%)	840 (34.75%)	903 (37.33%)	1012 (42.08%)	1161 (48.25%)	
Alcohol intake						< 0.001
No	7083 (73.08%)	1607 (66.27%)	1740 (71.66%)	1826 (75.64%)	1910 (78.76%)	
Yes	2609 (26.92%)	818 (33.73%)	688 (28.34%)	588 (24.36%)	515 (21.24%)	
Serum cotinine (ng/mL)	59.02 ± 128.79	73.95 ± 143.21	57.29 ± 126.00	53.68 ± 123.82	51.26 ± 119.85	< 0.001
Total Serum 25(OH)D (nmol/L)	62.50 ± 24.69	58.82 ± 25.18	62.11 ± 25.27	63.51 ± 24.56	65.56 ± 23.21	< 0.001
Total femur BMD (g/cm^2^)	0.98 ± 0.16	0.96 ± 0.16	0.96 ± 0.16	0.99 ± 0.16	1.00 ± 0.15	< 0.001
Femoral neck BMD (g/cm^2^)	0.84 ± 0.15	0.83 ± 0.15	0.83 ± 0.15	0.85 ± 0.15	0.86 ± 0.15	< 0.001
Trochanter BMD (g/cm^2^)	0.73 ± 0.13	0.72 ± 0.13	0.72 ± 0.13	0.74 ± 0.13	0.76 ± 0.13	< 0.001
Intertrochanter BMD (g/cm^2^)	1.16 ± 0.19	1.13 ± 0.19	1.14 ± 0.19	1.17 ± 0.18	1.19 ± 0.18	< 0.001
Total spine BMD (g/cm^2^)	1.03 ± 0.15	1.03 ± 0.16	1.02 ± 0.15	1.04 ± 0.15	1.04 ± 0.14	< 0.001

Dietary folate intake: Q1: < 260.5 μg/day; Q2: 260.5–359.5 μg/day; Q3: 360.0–492.0 μg/day; > 492.5 μg/day

Mean dietary folate intake: Total: 401 μg/day; Q1: 193.6 μg/day; Q2: 309.5 μg/day; Q3: 419.6 μg/day; Q4: 680.8 μg/day

Continuous variables were presented as mean ± SE. Categorical variables were presented as *n* (%). SD: standard deviation; MET: metabolic equivalent; BMD bone mineral density; Physical activity: Very Low PA (VLPA) (< 150 MET-min/week), Low PA (LPA) (150–960 MET-min/week), Medium PA (MPA) (961–1800MET-min/week) and High PA (HPA) (> 1800 MET-min/week)

The average dietary folate intake among the participants was quantified as 401.1 ± 207.9 μg/day. The BMD values ascertained for the total femur, femoral neck, trochanter, intertrochanter, and total spine were 0.98 g/cm^2^ (range: 0.82–1.14 g/cm^2^), 0.84 g/cm^2^ (range: 0.69–0.99 g/cm^2^), 0.73 g/cm^2^ (range: 0.60–0.86 g/cm^2^), 1.16 g/cm^2^ (range: 0.97–1.35 g/cm^2^), and 1.03 g/cm^2^ (range: 0.88–1.18 g/cm^2^), respectively.

A comparative analysis of the participants based on the quartiles of dietary folate intake revealed that the subjects within the higher quartiles (Q2–Q4) were generally younger than their counterparts in the first quartile (Q1). Additionally, these higher quartiles exhibited a larger proportion of female participants and non-Hispanic whites. They also reported a higher family income and demonstrated lower prevalence rates of smoking and drinking. An increase in physical activity was also observed among these subjects, along with elevated concentrations of serum 25(OH)D. Furthermore, a lower incidence of hypertension was recorded among the individuals in the superior quartiles (Q2–Q4).

### Relationship between dietary folate intake and BMD

Table 2 delineates the results of the linear correlation analysis between dietary folate intake and bone mineral density (BMD) at various skeletal sites, including the total femur, femoral neck, trochanter, intertrochanter, and lumbar spine. The unadjusted crude model indicated a positive correlation between dietary folate intake and BMD levels (*P* trend < 0.001 for all). This correlation persisted even after adjusting for potential confounders such as age, gender, and race. Individuals in the highest quartile (Q4) of dietary folate intake exhibited a potent correlation with elevated BMD levels relative to those in the first quartile, as demonstrated in the multivariable model. Dietary folate intake in the highest quartile was significantly associated with BMD at the total femur (*P* for trend = 0.003), femoral neck (*P* for trend = 0.016), intertrochanter (*P* for trend < 0.001), and lumbar spine (*P* for trend = 0.033). These results underscore a robust association between dietary folate intake and BMD at multiple skeletal sites.

**Table 2:** The association between dietary folate intake and BMD

Dietary folate intake (quintile)	Model 1		Model 2		Model 3		Model 4	
	*β* (95% CI)	*P* value	*β* (95% CI)	*P* value	*β* (95% CI)	*P* value	*β* (95% CI)	*P* value
Total femur, gm/cm^2^								
Q1	Reference		Reference		Reference		Reference	
Q2	0.006 (− 0.003, 0.015)	0.180	− 0.002 (− 0.010, 0.006)	0.636	− 0.002 (− 0.010, 0.006)	0.597	− 0.003 (− 0.010, 0.005)	0.519
Q3	0.032 (0.023, 0.041)	< 0.001	0.009 (0.001, 0.017)	0.024	0.008 (0.000, 0.016)	0.038	0.007 (− 0.001, 0.015)	0.092
Q4	0.046 (0.038, 0.055)	< 0.001	0.008 (− 0.000, 0.016)	0.051	0.011 (0.003, 0.019)	0.010	0.010 (0.002, 0.018)	0.017
*P* trend		< 0.001		0.011		0.001		0.003
Femoral neck, gm/cm^2^								
Q1	Reference		Reference		Reference		Reference	
Q2	0.001 (− 0.008, 0.009)	0.891	− 0.002 (− 0.009, 0.006)	0.681	− 0.002 (− 0.010, 0.005)	0.596	− 0.002 (− 0.010, 0.006)	0.595
Q3	0.022 (0.013, 0.030)	< 0.001	0.007 (− 0.001, 0.014)	0.080	0.005 (− 0.002, 0.013)	0.164	0.004 (− 0.004, 0.012)	0.285
Q4	0.030 (0.022, 0.039)	< 0.001	0.007 (− 0.001, 0.015)	0.071	0.008 (0.001, 0.016)	0.035	0.008 (− 0.000, 0.016)	0.052
*P* trend		< 0.001		0.022		0.009		0.016
Trochanter, gm/cm^2^								
Q1	Reference				Reference		Reference	
Q2	− 0.003 (− 0.011, 0.006)	0.528	− 0.005 (− 0.013, 0.004)	0.285	− 0.004 (− 0.013, 0.004)	0.316	− 0.004 (− 0.013, 0.005)	0.415
Q3	0.012 (0.004, 0.020)	0.005	0.003 (− 0.005, 0.012)	0.448	0.004 (− 0.005, 0.013)	0.354	0.005 (− 0.004, 0.014)	0.296
Q4	0.018 (0.009, 0.026)	< 0.001	0.003 (− 0.006, 0.011)	0.563	0.004 (− 0.005, 0.014)	0.332	0.005 (− 0.004, 0.014)	0.267
*P* trend		< 0.001		0.276		0.132		0.109
Intertrochanter, gm/cm^2^								
Q1	Reference		Reference		Reference		Reference	
Q2	0.006 (− 0.001, 0.013)	0.100	− 0.001 (− 0.008, 0.006)	0.839	− 0.001 (− 0.008, 0.006)	0.763	− 0.002 (− 0.009, 0.005)	0.666
Q3	0.027 (0.020, 0.035)	< 0.001	0.010 (0.003, 0.017)	0.005	0.008 (0.001, 0.015)	0.017	0.007 (− 0.000, 0.014)	0.050
Q4	0.040 (0.033, 0.047)	< 0.001	0.010 (0.003, 0.017)	0.005	0.011 (0.004, 0.018)	0.003	0.011 (0.003, 0.018)	0.004
*P* trend		< 0.001		0.001	0.000 (0.000, 0.000)	0.000		0.000
Total spine area, gm/cm^2^								
Q1	Reference		Reference		Reference		Reference	
Q2	0.005 (− 0.005, 0.016)	0.304	− 0.004 (− 0.014, 0.005)	0.360	− 0.004 (− 0.013, 0.005)	0.365	− 0.005 (− 0.014, 0.004)	0.291
Q3	0.034 (0.024, 0.044)	< 0.001	0.007 (− 0.002, 0.017)	0.140	0.007 (− 0.003, 0.016)	0.165	0.005 (− 0.005, 0.014)	0.320
Q4	0.050 (0.040, 0.060)	< 0.001	0.005 (− 0.005, 0.015)	0.318	0.009 (− 0.001, 0.018)	0.073	0.008 (− 0.002, 0.017)	0.128
*P* trend		< 0.001		0.117		0.016		0.033

*Model 1* Crude model

*Model 2* Adjusted for age, sex, race, PIR, and marital status

*Model 3* Adjusted for age, sex, race, PIR, marital status, BMI, hypertension, serum cotinine, physical activity, and alcohol use

*Model 4* Adjusted for age, sex, race, PIR, marital status, BMI, hypertension, serum cotinine, physical activity, alcohol use, and total vitamin D

*MET* Metabolic equivalent, *BMD* Bone mineral density

### Dose–response relation and threshold effect analysis via generalized additive model

**Fig. 2:**
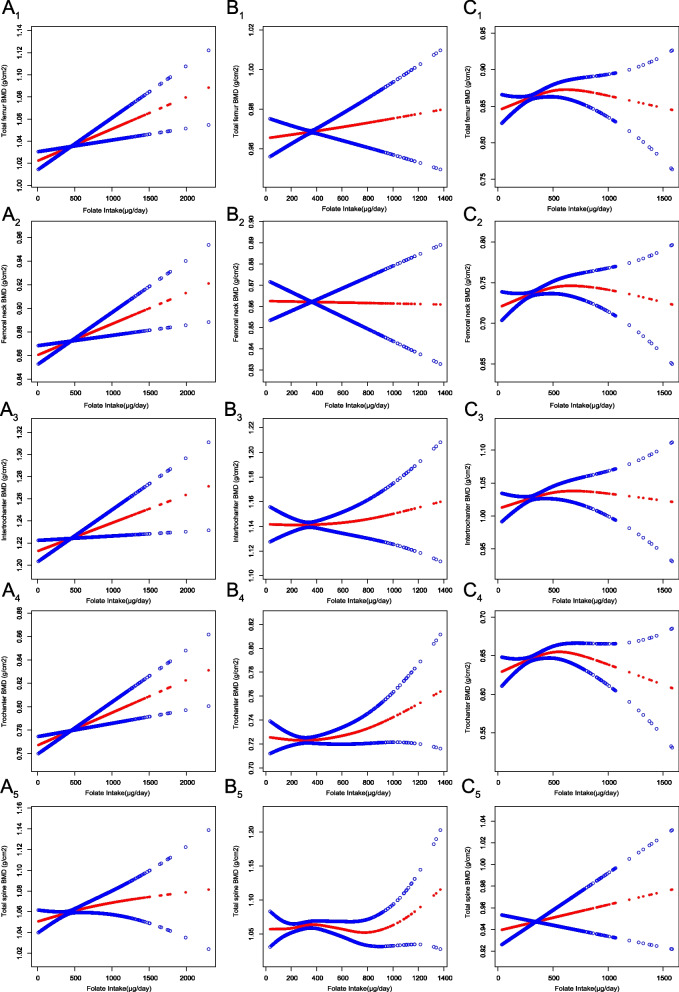
Relationship between dietary folate intake and BMD in postmenopausal women (**A**), premenopausal women (**B**) and men (**C**), 1 total femur, 2 femoral neck, 3 intertrochanter, 4 trochanter, 5 lumbar spine. The number of postmenopausal women = 2293; premenopausal women = 2516; men = 5030. Solid red line represents the smooth curve fit between variables according to GAM. Blue bands represent the 95% of confidence interval from the fit. Y-axis represents BMD content, and x-axis represents dietary folate intake. Models were adjusted for age, race, PIR, marital status, BMI, hypertension, serum cotinine, alcohol drinking status, PA, and 25(OH)D

Given distinct variations in factors influencing BMD among premenopausal women, postmenopausal women, and men, we sought to elucidate the nonlinear dose–response correlation among these groups, as delineated in Fig. 2A–C. The GAM smoothing curve fitting offers an expansive analytical framework by transforming coefficients into smooth functions of covariates, thereby efficaciously appraising interactions among factors without the pitfalls of underfitting or overfitting. This model surpasses the limitations of linear regression models, which typically postulate a specific functional relationship, such as linearity, between variables. In this context, the GAM does not enforce a preordained relationship between dietary folate intake and BMD, potentially leading to a superior fit [30].

Figure 2B and C exhibits a significant positive correlation between BMD and dietary folate intake in both male and premenopausal female populations, albeit with certain notable variances. Upon adjusting for potential confounding variables, total femur BMD in males displayed a greater sensitivity to folate intake compared to premenopausal females. However, a weak negative correlation was observed between folate intake and femoral neck BMD in premenopausal females, a pattern that diverges from other femoral regions. Moreover, lumbar spine BMD in premenopausal females indicated a significant increase only when daily folate intake surpassed 800 µg.

Figure 2A and Table 3 suggest that the positive correlation between dietary folate intake and lumbar spine BMD in postmenopausal women was not significant. Intriguingly, an inverted U-shaped relationship was perceived between folate intake and BMD in various femur regions in postmenopausal women. This observation was further scrutinized using a two-piecewise linear model to ascertain threshold effects. The inflection points for total femur BMD, femoral neck BMD, trochanter BMD, and intertrochanter BMD were determined to be 566, 569, 528, and 566 µg per day, respectively. For instance, for total femur BMD, a daily folate intake beyond 566 µg might precipitate a decline in BMD (Table 3).

**Table 3:** Threshold effect analysis of dietary folate intake and BMD in postmenopausal women

Threshold effect analysis of dietary folate intake and BMD in postmenopausal women	
	Adjusted HR (95% CI), *P* value
*Total femur BMD*	
Fitting by the standard linear model	0.00 (− 0.00, 0.00) 0.267
Fitting by the two-piecewise linear model	
Inflection point	566 μg/day
Dietary folate intake < Inflection point	0.00 (0.00, 0.00) 0.013
Dietary folate intake > Inflection point	− 0.00 (− 0.00, 0.00) 0.092
*P* for log-likelihood ratio	0.017
*Femoral neck BMD*	
Fitting by the standard linear model	0.00 (− 0.00, 0.00) 0.505
Fitting by the two-piecewise linear model	
Inflection point	569 μg/day
Dietary folate intake < Inflection point	0.00 (− 0.00, 0.00) 0.053
Dietary folate intake > Inflection point	− 0.00 (− 0.00, 0.00) 0.115
*P* for log-likelihood ratio	0.040
*Trochanter BMD*	
Fitting by the standard linear model	0.00 (− 0.00, 0.00) 0.384
Fitting by the two-piecewise linear model	
Inflection point	528 μg/day
Dietary folate intake < Inflection point	0.00 (0.00, 0.00) 0.004
Dietary folate intake > Inflection point	− 0.00 (− 0.00, − 0.00) 0.024
*P* for log-likelihood ratio	0.003
*Intertrochanter BMD*	
Fitting by the standard linear model	0.00 (− 0.00, 0.00) 0.337
Fitting by the two-piecewise linear model	
Inflection point	566 μg/day
Dietary folate intake < Inflection point	0.00 (0.00, 0.00) 0.042
Dietary folate intake > Inflection point	− 0.00 (− 0.00, 0.00) 0.189
*P* for log-likelihood ratio	0.057
*Total spine BMD*	
Fitting by the standard linear model	0.00 (− 0.00, 0.00) 0.350
Fitting by the two-piecewise linear model	
Inflection point	249.5 μg/day
Dietary folate intake < inflection point	0.00 (− 0.00, 0.00) 0.446
Dietary folate intake > inflection point	0.00 (− 0.00, 0.00) 0.633
*P* for log-likelihood ratio	0.564

Adjusted for age, gender, race, marital status, PIR, BMI, alcohol consumption, PA, history of hypertension, serum cotinine and serum vitamin D

*BMD* Bone mineral density

### Further subgroup analysis

**Fig. 3:**
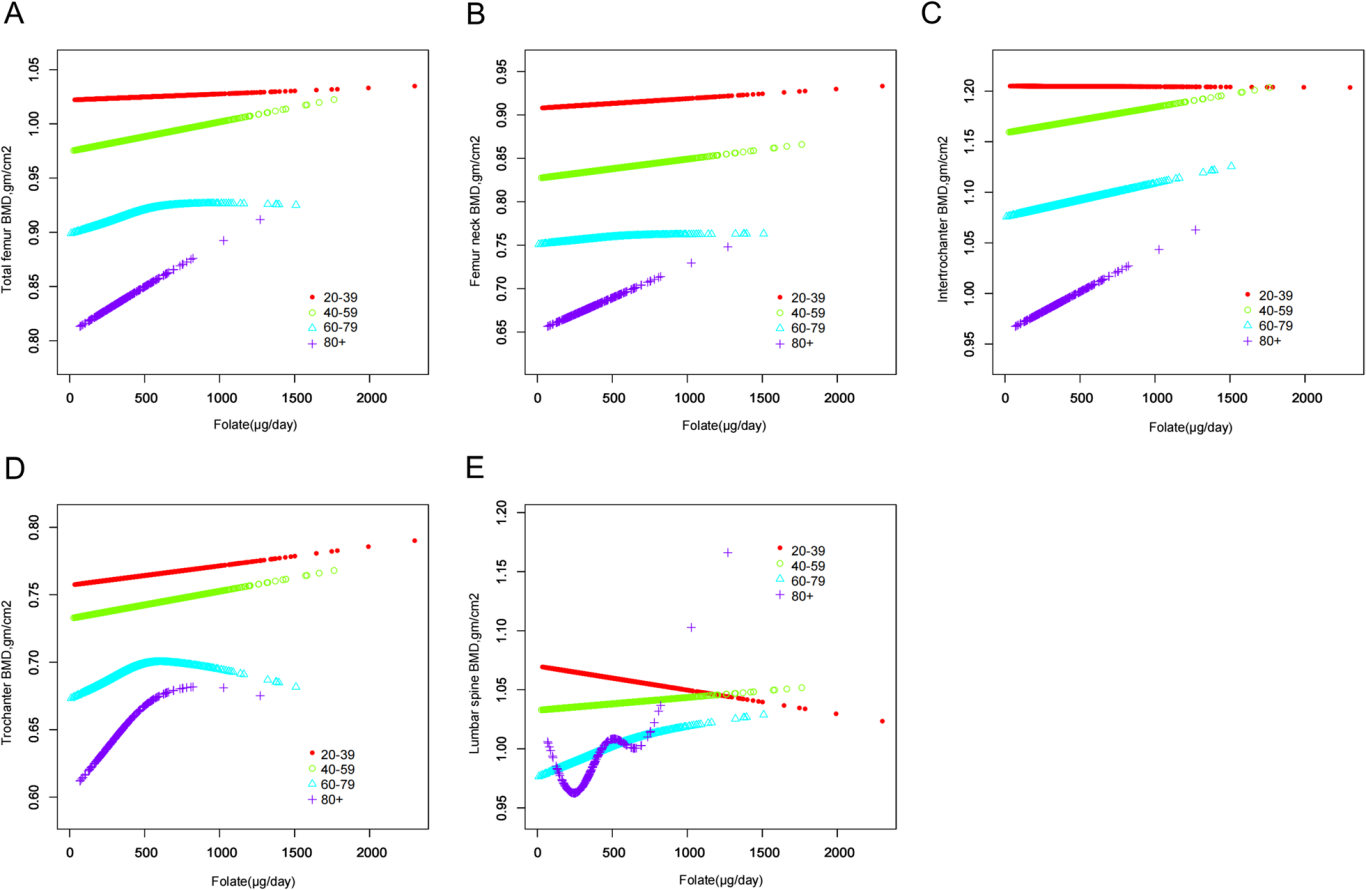
Relationship between dietary folate intake and BMD in different ages, **A** total femur, **B** femoral neck, **C** intertrochanter, **D** trochanter, **E** lumbar spine. The red line represents 20–39 years old; the green line represents 40–59 years old; the blue line represents 59–79 years old; and the purple line represents greater than or equal to 80 years old. Y-axis represents BMD content, and x-axis represents dietary folate intake. Models were adjusted for age, gender, race, PIR, marital status, BMI, hypertension, serum cotinine, alcohol drinking status, PA, and serum 25(OH)D

Subgroup analysis, stratified by age as shown in Fig. 3, unveiled a significant positive correlation between dietary folate intake and BMD levels in participants aged 80 years and older (*P *= 0.014). This intriguing finding suggests that this demographic may derive a greater benefit from increasing their dietary folate intake to maintain optimal bone health. In contrast, spinal BMD displayed a negative correlation in individuals aged between 20 and 40 years, the underlying reasons for which warrant further investigation.

**Fig. 4:**
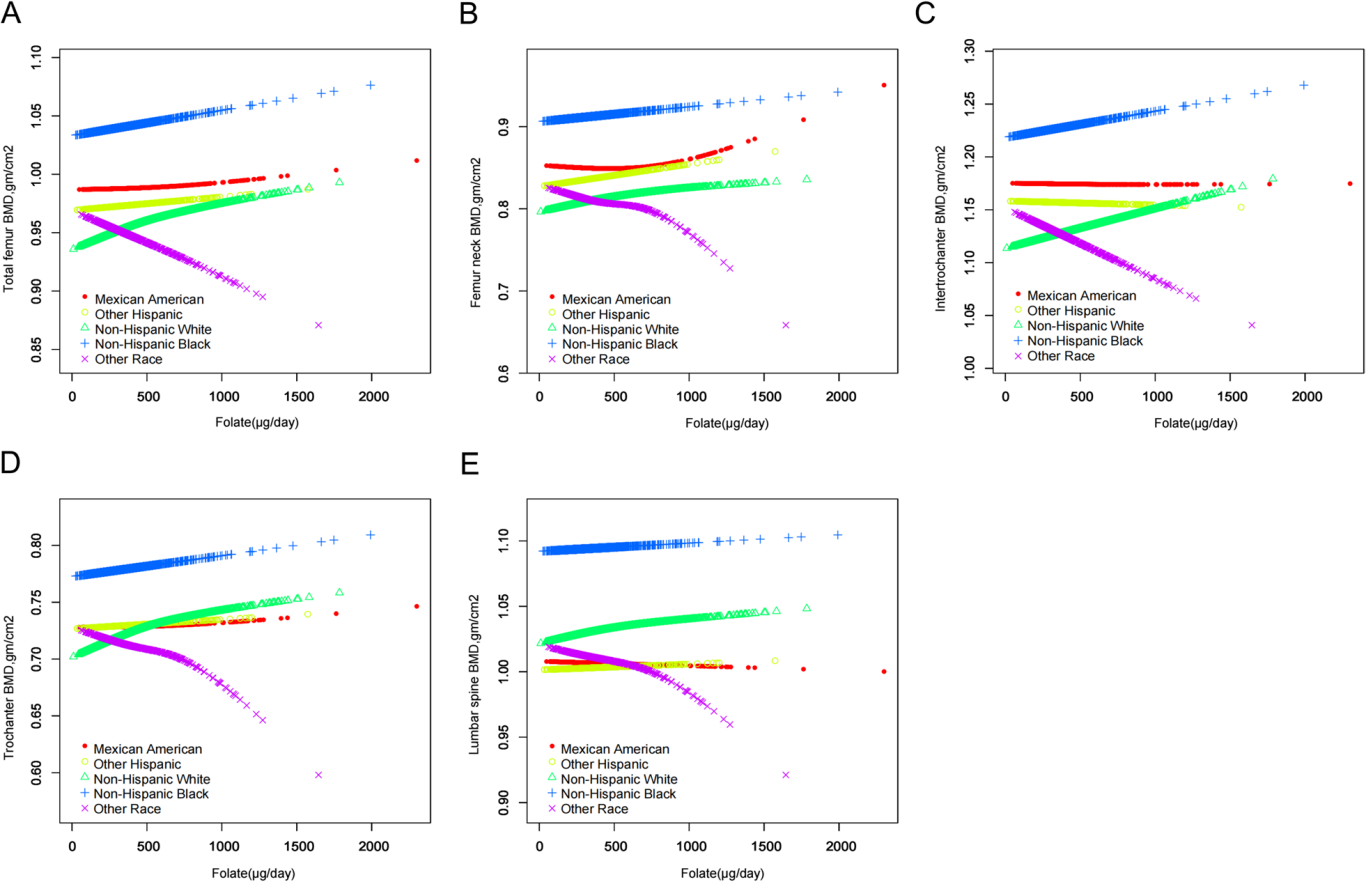
Relationship between dietary folate intake and BMD in different races, **A** total femur, **B** femoral neck, **C** intertrochanteric, **D** trochanter, **E** lumbar spine. The red line represents Mexican American, the yellow line represents other Hispanic, the green line represents non-Hispanic white, the blue line represents non-Hispanic black, and the purple line represents Other Race—Including Multi-Racial. Y-axis represents BMD content, and x-axis represents dietary folate intake. Models were adjusted for age, gender, PIR, marital status, BMI, hypertension, serum cotinine, alcohol drinking status, PA, and serum 25(OH)D

The subgroup analysis stratified by ethnicity is elucidated in Fig. 4. A notable finding is the positive relationship between increased dietary folate intake and elevated BMD across ethnic groups, including non-Hispanic whites, blacks, Mexican Americans, and other Hispanics. The most pronounced BMD elevations were observed among non-Hispanic whites, suggesting potential benefits from adequate daily folate supplementation in this group. Conversely, the “Other Race—Including Multi-Racial” groups exhibited an inverse relationship between folate intake and BMD, although this negative trend may be unreliable due to the small sample size and inclusion of multiple races. Remarkably, blacks exhibited higher BMD levels than other ethnic groups across all regions, emphasizing the necessity to consider ethnic-specific differences in the pathogenesis and management of bone health disorders.

**Fig. 5:**
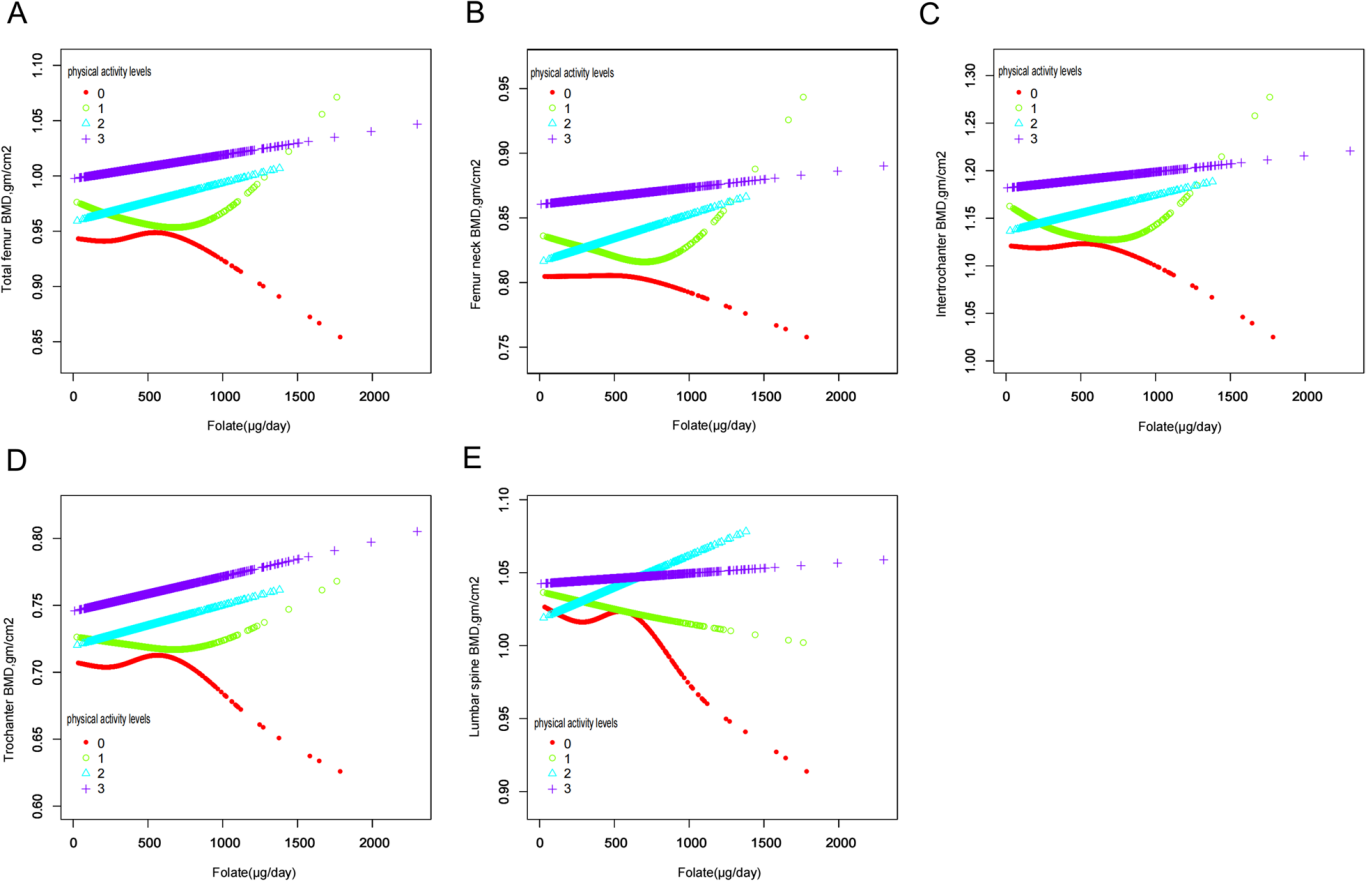
Relationship between dietary folate intake and BMD in people with different PA levels, **A** total femur, **B** femoral neck, **C** intertrochanter, **D** trochanter, **E** lumbar spine. The red line represents VLPA, the green line represents LPA, the blue line represents MPA, and the purple line represents HPA. Y-axis represents BMD content, and x-axis represents dietary folate intake. Models were adjusted for age, gender, race, PIR, marital status, BMI, hypertension, serum cotinine, alcohol drinking status, PA, and serum 25(OH)D

Figure 5 displays the results of the subgroup analysis stratified by physical activity level. The multivariate regression analysis, which accounted for all covariates, showed a positive relationship between dietary folate intake and BMD levels in individuals with high (HPA) and moderate (MPA) physical activity. However, among individuals with low physical activity (LPA), a negative correlation between folate intake and BMD levels in the lumbar spine region was observed. Furthermore, participants with very low physical activity (VLPA) exhibited a nonlinear decreasing trend in BMD levels with increasing folate intake. These results have significant clinical implications, suggesting that the interaction between folate intake and BMD levels is influenced by physical activity levels. Consequently, individuals with LPA and VLPA may require additional interventions to optimize their bone health, such as increasing physical activity levels and/or adjusting dietary folate intake.

**Fig. 6:**
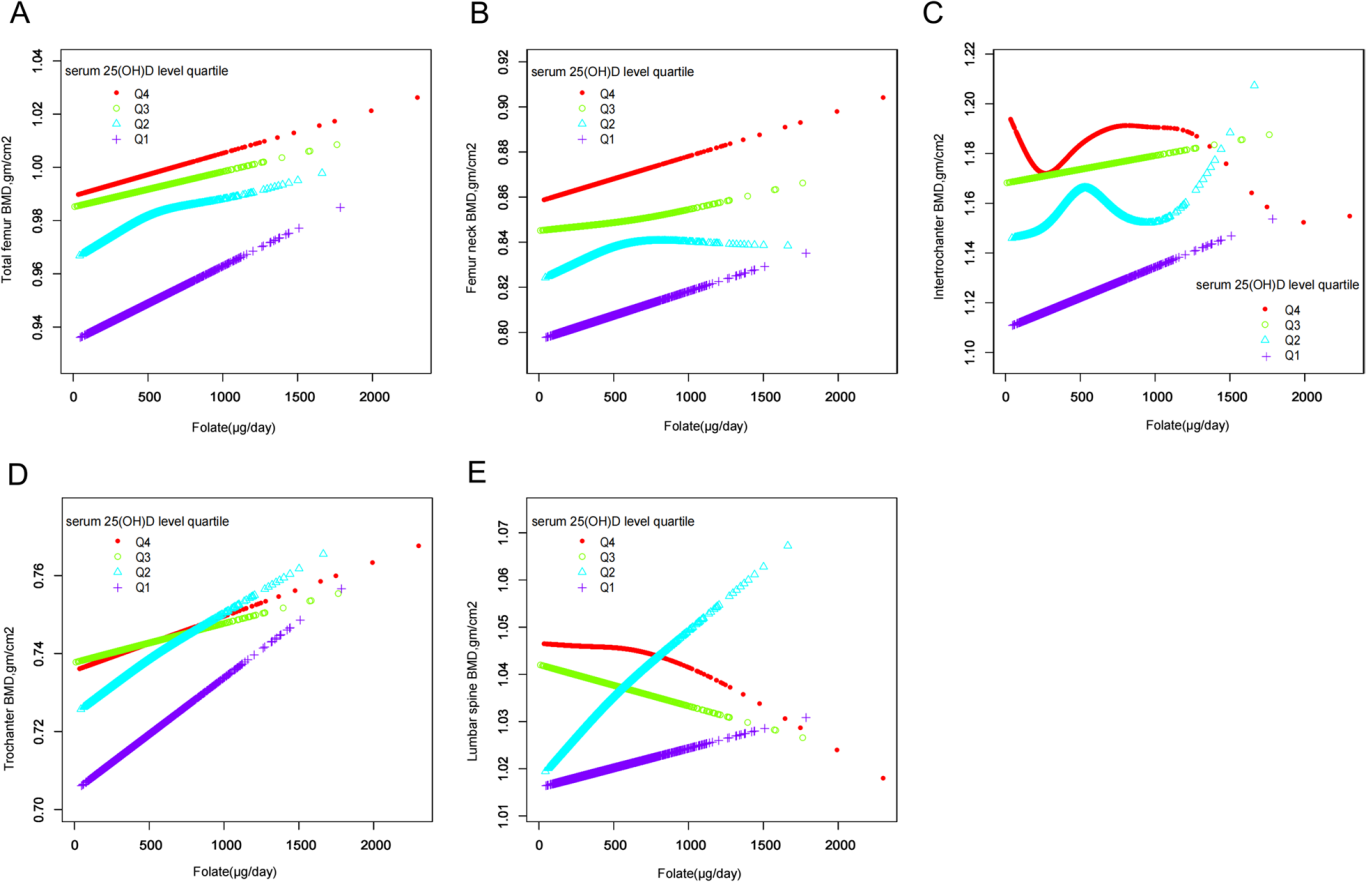
Relationship between dietary folate intake and BMD in people with different serum 25(OH)D, **A** total femur, **B** femoral neck, **C** intertrochanter, **D** trochanter, **E** lumbar spine. The red line represents 25(OH)D levels greater than 76.5 nmol/L, the green line represents between 25(OH)D levels 60.4 and 76.5 nmol/L, the blue line represents 25(OH)D levels between 44.7 and 60.4 nmol/L, and the purple line represents 25(OH)D levels less than 44.7 nmol/L. Y-axis represents BMD content, and x-axis represents dietary folate intake. Models were adjusted for age, gender, race, PIR, marital status, BMI, hypertension, serum cotinine, alcohol drinking status, PA, and serum 25(OH)D

Figure 6 presents the results of the subgroup analysis stratified by 25(OH)D levels. After adjusting for other confounding variables, the findings indicate that an elevated intake of folate is associated with increased BMD levels in the total femur, femoral neck, and intertrochanteric region. Interestingly, participants with serum 25(OH)D levels greater than 76.5 nmol/L (Q4) and between 60.4 and 76.5 nmol/L (Q3) displayed a negative association between folate intake and BMD levels in the lumbar spine. Further research is merited to elucidate the underlying mechanisms of this association. Importantly, the lumbar spine results suggest a potential interaction effect between serum 25(OH)D concentrations and folate intake, as BMD levels in the high 25(OH)D group exhibit an inverse correlation with folate intake. This finding underscores the need for further investigation into the complex interplay between dietary folate intake, serum 25(OH)D levels, and bone health.

## Discussion

This study provides a comprehensive investigation into the intricate relationship between dietary folate intake and BMD levels. The study unveils a substantial and pervasive positive correlation between dietary folate intake and BMD levels. Moreover, our subgroup analyses suggest that this correlation is influenced by several factors including menopausal status, sex, serum 25-hydroxyvitamin D [25(OH)D] levels, physical activity, age, and race, thereby enriching our understanding of the complex interplay between dietary folate intake and BMD levels.

Both low BMD and osteoporosis are pervasive public health issues, leading to considerable economic burden and diminished quality of life. Folate, also recognized as vitamin B9, plays a crucial role in human health and carries multiple benefits for various physiological systems, including the nervous and skeletal systems [37–39]. It has been demonstrated that folate intervention can significantly enhance bone microstructure in high-fat diet (HFD) mice [40, 41]. Folate supplementation resulted in a decrease in osteoclast count in HFD-fed mice while augmenting the number of adipocytes, suggesting that folate may regulate lipid metabolism and impact the onset of osteoporosis [11].

Long-term folate deficiency can instigate obesity, lipid metabolism disorders, and glucose metabolism disorders, while appropriate folate intake can mitigate the risk of dyslipidemia [42, 43]. Moreover, osteoporosis and osteoporotic fractures have been linked to folate deficiency and hyperhomocysteinemia [44–46], a metabolic product of the essential amino acid methionine. Folate serves as a critical coenzyme for homocysteine degradation through remethylation and transsulfuration pathways, and hence, folate deficiency can cause a surge in serum homocysteine concentration (hyperhomocysteinemia). Furthermore, folate plays an essential role in reducing oxidative stress and protein methylation [47], which in turn decreases the incidence of osteoporosis by directly eliminating and stimulating the expression of antioxidant enzymes [10].

The current body of research on the relationship between dietary folate intake and BMD is somewhat limited. While a number of studies have investigated the association between other B vitamins, homocysteine, and bone health, few have specifically examined the association between dietary folate intake and BMD in the general population. Furthermore, some of these studies may be constrained by inadequate sample sizes, limiting the capacity to draw definitive conclusions about the relationship between dietary folate intake and BMD in the general population [48–50].

Furthermore, in certain populations, such as those with Very Low Physical Activity (VLPA), the relationship between dietary folate intake and BMD levels demonstrated an inverse correlation. Various explanations for this finding exist. Notably, physical exercise has been shown to counteract, delay, and mitigate the adverse effects of osteoporosis [51]. Exercise can also decrease bone resorption [52] and augment BMD [53]. In premenopausal women and individuals aged 20–40, an increase in folate intake was associated with a decrease in lumbar BMD levels. However, the underlying reasons and mechanisms for this phenomenon remain unknown.

Folate can promote the growth and differentiation of bone cells, increase bone formation, and reduce the occurrence of osteoporosis. This is because folate plays a pivotal role in DNA synthesis and repair, gene expression regulation, and consequently, bone cell growth and differentiation [54]. However, in some specific populations, an increase in folate intake does not necessarily lead to an increase in BMD. This may be because excessive intake of folate can interfere with the body's absorption of other vital nutrients, such as calcium, magnesium, and 25(OH)D, thereby affecting BMD. Therefore, the effect of folate on BMD may be influenced by the overall nutrient balance in the body.

This research boasts several noteworthy strengths. Firstly, it utilizes a large, nationally representative dataset, gathered through standardized protocols, which minimizes potential biases. Secondly, the study diligently categorizes folate intake levels into quartiles and tests for linear trends, thereby assuring robust and accurate data interpretation. Thirdly, this research effectively accounts for potential confounding variables while evaluating the correlation between dietary folate intake and BMD levels, as well as the variations in osteoporosis risk among different populations stratified by menopausal status, gender, 25(OH)D, physical activity, age, and race. Additionally, the study employs a versatile generalized additive model to explore potential nonlinear trends and conducts a threshold effects analysis to investigate the optimal dietary folate intake for postmenopausal women. Given the apparent inverted U-shaped relationship between BMD in the femur of postmenopausal women and folate intake, the study suggests that daily dietary folate intake for postmenopausal women should not exceed 528–569 µg per day for optimal bone health.

Notwithstanding our study's valuable insights, there are some potential limitations that should be acknowledged. Firstly, as a cross-sectional analysis, the evidence for causality may be limited due to the lack of temporal sequence. Thus, further longitudinal studies are warranted to explore the potential causality between dietary folate intake and BMD levels. Secondly, the data collected from self-reported questionnaires and interviews may introduce recall bias, which may affect the accuracy of the data. Thirdly, although we adjusted for several potential confounding variables, some other unmeasured confounding factors may have influenced our results. Therefore, future studies should consider these factors and conduct more in-depth analyses to confirm our findings.

## Conclusions

In conclusion, this study provides valuable insights into the correlation between dietary folate intake and BMD levels in the general US population. It underscores the significance of dietary folate intake for bone health and highlights the potential implications of personalized dietary interventions for the prevention and management of osteoporosis. However, further research is warranted to elucidate the complex mechanisms underlying the relationship between dietary folate intake and BMD and to validate the findings in diverse populations and settings.

## Data Availability

The data used in this study can be downloaded for free in NHANES.

## Abbreviations

NHANES: National Health and Nutrition Examination Survey
BMD: Bone mineral density
DXA: Dual-energy X-ray absorptiometry
GAM: Generalized additive model
CI: Confidence interval
USD: US dollar
GBP: Great Britain Pound
AMPK: AMP-activated protein kinase
HFD: High-fat diet
NCHS: National Center for Health Statistics
USDA: United States Department of Agriculture
AMPM: The automated multiple-pass method
BMI: Body mass index
PA: Physical activity
25(OH)D: 25-hydroxyvitamin D
ANOVA: Analysis of variance
HPA: High physical activity
MPA: Medium physical activity
LPA: Low physical activity
VLPA: Very low physical activity

## References

[1] Lorentzon M, Nilsson AG, Johansson H, Kanis JA, Mellström D, Sundh D (2019). Extensive undertreatment of osteoporosis in older Swedish women. Osteoporos Int.

[2] Johnston CB, Dagar M (2020). Osteoporosis in older adults. Med Clin North Am.

[3] Hernlund E, Svedbom A, Ivergård M, Compston J, Cooper C, Stenmark J, McCloskey EV, Jönsson B, Kanis JA (2013). Osteoporosis in the European Union: medical management, epidemiology and economic burden. A report prepared in collaboration with the International Osteoporosis Foundation (IOF) and the European Federation of Pharmaceutical Industry Associations (EFPIA). Arch Osteoporos.

[4] Office of the Surgeon G: Reports of the Surgeon General (2004). Bone health and osteoporosis: a report of the surgeon general.

[5] Field MS, Stover PJ (2018). Safety of folic acid. Ann N Y Acad Sci.

[6] Schiavone S, Colaianna M, Curtis L (2015). Impact of early life stress on the pathogenesis of mental disorders: relation to brain oxidative stress. Curr Pharm Des.

[7] Filaire E, Toumi H (2012). Reactive oxygen species and exercise on bone metabolism: friend or enemy?. Joint Bone Spine.

[8] Manolagas SC (2010). From estrogen-centric to aging and oxidative stress: a revised perspective of the pathogenesis of osteoporosis. Endocr Rev.

[9] Rached MT, Kode A, Xu L, Yoshikawa Y, Paik JH, Depinho RA, Kousteni S (2010). FoxO1 is a positive regulator of bone formation by favoring protein synthesis and resistance to oxidative stress in osteoblasts. Cell Metab.

[10] Kimball JS, Johnson JP, Carlson DA (2021). Oxidative stress and osteoporosis. J Bone Joint Surg Am.

[11] He H, Zhang Y, Sun Y, Zhang Y, Xu J, Yang Y, Chen J (2021). Folic acid attenuates high-fat diet-induced osteoporosis through the ampk signaling pathway. Front Cell Dev Biol.

[12] Damani JJ, De Souza MJ, VanEvery HL, Strock NCA, Rogers CJ (2022). The role of prunes in modulating inflammatory pathways to improve bone health in postmenopausal women. Adv Nutr.

[13] Martínez-Ramírez MJ, Palma Pérez S, Delgado-Martínez AD, Martínez-González MA, De la Fuente AC, Delgado-Rodríguez M (2007). Vitamin C, vitamin B12, folate and the risk of osteoporotic fractures. A case-control study. Int J Vitam Nutr Res.

[14] Herrmann M, Peter Schmidt J, Umanskaya N, Wagner A, Taban-Shomal O, Widmann T, Colaianni G, Wildemann B, Herrmann W (2007). The role of hyperhomocysteinemia as well as folate, vitamin B(6) and B(12) deficiencies in osteoporosis: a systematic review. Clin Chem Lab Med.

[15] Akpolat V, Bilgin HM, Celik MY, Erdemoglu M, Isik B (2013). An evaluation of nitric oxide, folate, homocysteine levels and lipid peroxidation in postmenopausal osteoporosis. Adv Clin Exp Med.

[16] Bailey RL, van Wijngaarden JP (2015). The role of B-vitamins in bone health and disease in older adults. Curr Osteoporos Rep.

[17] Haliloglu B, Aksungar FB, Ilter E, Peker H, Akin FT, Mutlu N, Ozekici U (2010). Relationship between bone mineral density, bone turnover markers and homocysteine, folate and vitamin B12 levels in postmenopausal women. Arch Gynecol Obstet.

[18] Lee K, Lim S, Park H, Woo HY, Chang Y, Sung E, Jung HS, Yun KE, Kim CW, Ryu S (2020). Subclinical thyroid dysfunction, bone mineral density, and osteoporosis in a middle-aged Korean population. Osteoporos Int.

[19] Bendavid EJ, Shan J, Barrett-Connor E (1996). Factors associated with bone mineral density in middle-aged men. J Bone Miner Res.

[20] Bailey RL, Looker AC, Lu Z, Fan R, Eicher-Miller HA, Fakhouri TH, Gahche JJ, Weaver CM, Mills JL (2015). B-vitamin status and bone mineral density and risk of lumbar osteoporosis in older females in the United States. Am J Clin Nutr.

[21] Cagnacci A, Bagni B, Zini A, Cannoletta M, Generali M, Volpe A (2008). Relation of folates, vitamin B12 and homocysteine to vertebral bone mineral density change in postmenopausal women. A five-year longitudinal evaluation. Bone.

[22] Zhang Y, Jia X, Liu X, An W, Li J, Zhang W (2022). Relationship between different body composition and bone mineral density in Qinhuangdao city. Rev Assoc Med Bras.

[23] Cai S, Fan J, Zhu L, Ye J, Rao X, Fan C, Zhong Y, Li Y (2020). Bone mineral density and osteoporosis in relation to all-cause and cause-specific mortality in NHANES: a population-based cohort study. Bone.

[24] Johnson CL, Paulose-Ram R, Ogden CL, Carroll MD, Kruszon-Moran D, Dohrmann SM, Curtin LR (2013). National health and nutrition examination survey: analytic guidelines, 1999–2010. Vital Health Stat.

[25] Pannucci TE, Thompson FE, Bailey RL, Dodd KW, Potischman N, Kirkpatrick SI, Alexander GL, Coleman LA, Kushi LH, Groesbeck M (2018). Comparing reported dietary supplement intakes between two 24-hour recall methods: the automated self-administered 24-hour dietary assessment tool and the interview-administered automated multiple pass method. J Acad Nutr Diet.

[26] DiGrande L, Pedrazzani S, Kinyara E, Hymes M, Karns S, Rhodes D, Moshfegh A. RTI Press Methods Report Series. In: Field Interviewer–Administered Dietary Recalls in Participants’ Homes: A Feasibility Study Using the US Department of Agriculture’s Automated Multiple-Pass Method. Research Triangle Park (NC): RTI Press© 2021 Research Triangle Institute. All rights reserved.; 2021.

[27] Foster E, Lee C, Imamura F, Hollidge SE, Westgate KL, Venables MC, Poliakov I, Rowland MK, Osadchiy T, Bradley JC (2019). Validity and reliability of an online self-report 24-h dietary recall method (Intake24): a doubly labelled water study and repeated-measures analysis. J Nutr Sci.

[28] Jiang H, Wang K, Zhang H, Yang B, Mao W, Chen M, Zhou S (2023). Physical activity can influence the relationship between ethylene oxide and risk of kidney stones: a cross-sectional study from the NHANES 2013–2016. Environ Sci Pollut Res.

[29] Zhang Y, Zhao C, Zhang H, Chen M, Meng Y, Pan Y, Zhuang Q, Zhao M (2023). Association between serum soluble α-klotho and bone mineral density (BMD) in middle-aged and older adults in the United States: a population-based cross-sectional study. Aging Clin Exp Res.

[30] Perperoglou A, Sauerbrei W, Abrahamowicz M, Schmid M (2019). A review of spline function procedures in R. BMC Med Res Methodol.

[31] Lin L, Chen CZ, Yu XD (2013). The analysis of threshold effect using Empower Stats software. Zhonghua Liu Xing Bing Xue Za Zhi.

[32] Wen Z, Li X (2023). Association between weight-adjusted-waist index and female infertility: a population-based study. Front Endocrinol.

[33] Reginster JY, Burlet N (2006). Osteoporosis: a still increasing prevalence. Bone.

[34] Jones G, Nguyen T, Sambrook PN, Kelly PJ, Gilbert C, Eisman JA (1994). Symptomatic fracture incidence in elderly men and women: the Dubbo Osteoporosis Epidemiology Study (DOES). Osteoporos Int.

[35] Hudec SM, Camacho PM (2013). Secondary causes of osteoporosis. Endocr Pract.

[36] Tang Y, Yi Q, Wang S, Xia Y, Geng B (2022). Normal concentration range of blood mercury and bone mineral density: a cross-sectional study of National Health and Nutrition Examination Survey (NHANES) 2005–2010. Environ Sci Pollut Res Int.

[37] Rondanelli M, Opizzi A, Berzero M (2007). Focus on folic acid benefits. Minerva Gastroenterol Dietol.

[38] Pfeiffer CM, Gunter EW, Miller DT (1999). Folic acid fortification. N Engl J Med.

[39] Lucock M, Yates Z (2009). Folic acid fortification: a double-edged sword. Curr Opin Clin Nutr Metab Care.

[40] Lin C, Yu S, Jin R, Xiao Y, Pan M, Pei F, Zhu X, Huang H, Zhang Z, Chen S (2019). Circulating miR-338 cluster activities on osteoblast differentiation: potential diagnostic and therapeutic targets for postmenopausal osteoporosis. Theranostics.

[41] Li J, Li X, Liu D, Hamamura K, Wan Q, Na S, Yokota H, Zhang P (2019). eIF2α signaling regulates autophagy of osteoblasts and the development of osteoclasts in OVX mice. Cell Death Dis.

[42] Dehkordi EH, Sedehi M, Shahraki ZG, Najafi R (2016). Effect of folic acid on homocysteine and insulin resistance of overweight and obese children and adolescents. Adv Biomed Res.

[43] Gargari BP, Aghamohammadi V, Aliasgharzadeh A (2011). Effect of folic acid supplementation on biochemical indices in overweight and obese men with type 2 diabetes. Diabetes Res Clin Pract.

[44] Yazdanpanah N, Zillikens MC, Rivadeneira F, de Jong R, Lindemans J, Uitterlinden AG, Pols HAP, van Meurs JBJ (2007). Effect of dietary B vitamins on BMD and risk of fracture in elderly men and women: the Rotterdam study. Bone.

[45] McLean RR, Jacques PF, Selhub J, Tucker KL, Samelson EJ, Broe KE, Hannan MT, Cupples LA, Kiel DP (2004). Homocysteine as a predictive factor for hip fracture in older persons. N Engl J Med.

[46] van Meurs JB, Dhonukshe-Rutten RA, Pluijm SM, van der Klift M, de Jonge R, Lindemans J, de Groot LC, Hofman A, Witteman JC, van Leeuwen JP (2004). Homocysteine levels and the risk of osteoporotic fracture. N Engl J Med.

[47] Herrmann W, Herrmann M, Obeid R (2007). Hyperhomocysteinaemia: a critical review of old and new aspects. Curr Drug Metab.

[48] Fratoni V, Brandi ML (2015). B vitamins, homocysteine and bone health. Nutrients.

[49] Holstein JH, Herrmann M, Splett C, Herrmann W, Garcia P, Histing T, Graeber S, Ong MF, Kurz K, Siebel T (2009). Low serum folate and vitamin B-6 are associated with an altered cancellous bone structure in humans. Am J Clin Nutr.

[50] Swart KM, van Schoor NM, Lips P (2013). Vitamin B12, folic acid, and bone. Curr Osteoporos Rep.

[51] Gauthier A, Kanis JA, Jiang Y, Martin M, Compston JE, Borgström F, Cooper C, McCloskey EV (2011). Epidemiological burden of postmenopausal osteoporosis in the UK from 2010 to 2021: estimations from a disease model. Arch Osteoporos.

[52] Senderovich H, Kosmopoulos A (2018). An insight into the effect of exercises on the prevention of osteoporosis and associated fractures in high-risk individuals. Rambam Maimonides Med J.

[53] Eleftheriou KI, Rawal JS, Kehoe A, James LE, Payne JR, Skipworth JR, Puthucheary ZA, Drenos F, Pennell DJ, Loosemore M (2012). The Lichfield bone study: the skeletal response to exercise in healthy young men. J Appl Physiol (1985).

[54] Duthie SJ (1999). Folic acid deficiency and cancer: mechanisms of DNA instability. Br Med Bull.

